# A bibliometric study of the movement disorder field by analyzing classic citation data on publication

**Published:** 2018-01-05

**Authors:** Kaveh Shafiei, Mahdiyeh Khazaneha

**Affiliations:** 1 Neurology Research Centre, Kerman University of Medical Sciences, Kerman, Iran; 2 Department of Knowledge and Information Sciences, School of Management and Information Science, Kerman University of Medical Sciences, Kerman, Iran

**Keywords:** Bibliometric Analysis, Movement Disorders, Medical Journalism

## Abstract

**Background:** The extent to which a publication attracts scientific attention by virtue of its focus, provides clues about the trend and volume of scientific production in a particular field. Bibliometric analysis is a method to quantify aspects of a specific research area. This article focused on publication on the movement disorders.

**Methods:** The bibliometric data on movement disorder were retrieved in relation to set of keywords from the Thomson Reuters Web of Science (WOS) available by January 2017. As many bibliometric statistics, such as citation indexes change during time, it was decided to compare two successive 5-years periods, 2007-2011 and 2012-2016. In addition, the citation classics publications with more than 100 times cited were taken into consideration.

**Results:** In total, the number of movement disorder papers rose from 49,444 to 61,942. Clinical neurology was the most studied WOS subfield at 35.6%. About 12.0% of these studies were published by the Movement Disorders Journal. Original papers accounted for 63.7% of publications. The United States of America was the leading country as the source of these publications followed by China. University College London (UCL) was associated more than any other university regarding these publications whereas the National Institute of Health (NIH) was the major source of funding. The number of papers with more than 100 citations totaled 87, out of which only one paper had more than 400 citations.

**Conclusion:** This study demonstrates that the total number of publications in movement disorders has increased remarkably during the past decade.

## Introduction

It goes without saying that for pundits in the medical profession anticipating success of a publication in procuring peers attention, is of crucial importance in directing their research to a specific field. Bibliometric analysis is of great value to measure and quantify the volume and pertinence of publication in the medical field by identifying citation indexes.^1^ Variables such as frequency of each article, author, or journal cited can be extracted by this method. Furthermore, publications can be ranked and researchers with highest impact within a specific research field can be identified. The citation indexes not only has significant implications for research and education, but also quantify the impact of publication on scientific community.^2^^-^^5^

This is done by two different bibliometric approaches: 1. setting citation thresholds, for example listing all papers cited with more than 400 or 100 times, and 2. listing most frequent cited papers. Recently, researchers prefer information on the most time-cited paper rather than setting a threshold for extracting and consulting list of publications.^5^

Whereas many still doubt the significance of bibliometric analysis, this method is likely to provide reliable quantitative measurements to determine the impact of an article or journal on scientific community.^2^^,^^6^

Bibliometric analysis of scientific publication could easily be extracted from online resources such as the Thomson Reuter’s Web of Science (WOS). This data base indexes more than 10000 high-impact journals in sciences fields.^2^

Few bibliometric analyses on the movement disorders field has been accomplished so far. As scientific literature on movement disorder is rapidly accumulating, bibliometric analysis of this field area by publication is of particular importance.^7^^-^^10^

It this short communication, we compared statistics of two successive 5-years periods (2007-2011 and 2012-201) in order to establish the trend in scientific output.

## Materials and Methods

WOS database has great coverage of high quality scientific papers, and provides citation analysis. 

The strategy of the current study was to search the following keywords in the topic search box on the WOS database, extracted from Medical Subject Headings (MeSH): Parkinson’s disease, akathisia, dyskinesia, dystonia, tremor, parkinsonism, tic, myoclonus, and chorea. Then, the retrieved publication were analyzed for authorship, WOS category of research field area, document type, source title (publisher), country (based on first author affiliation), founding agency, and affiliated university during the two 5-year time periods (2007-2011 and 2012-2016). 

Statistics of the citation classics publication between 2012 and 2016 also were taken into account in the second part of our bibliometric analysis. Publications cited more than 100 times were considered for citation classic. Similarly, title and abstract of the publication cited more than 100 times were studied, and related publications were selected for further analysis.

## Results

An observational bibliometric analysis was undertaken on all and the citation classic publications in movement disorders between two 5-year time periods, 2007-2011 and 2012-2016. English was the overwhelming language of the papers written accounting for almost 98% of all publications. 

Between 2007-2011 and 2012-2016 periods, the total number of scientific papers increased from 49444 to 61942. Clinical neurology was the most studied WOS field (40.2% and 35.5% during the two periods, respectively). Original papers were dominant (61.4% and 63.7% during the two periods, respectively). Movement Disorders Journal of Movement Disorder Society (MDS) published most of the papers (12.1% and 13.4% during the two periods, respectively) followed by Public Library of Science (PLOS) one (0.7% and 2.6% during the two periods, respectively), and in turn by Parkinsonism-Related Disorders Journal (2.4% and 2.2% during the two periods, respectively), Journal of Neurology (1.3% and 1.7% during the two periods, respectively) and European Journal of Neurology (2.0% and 1.7% during the two periods, respectively). Number of publication in movement disorders article significantly increases from 379 to 1664 (almost three fold) in the PLOS one, a peer-reviewed open access scientific journal published by the PLOS.

United States was contributing most of the publications in movement disorders (29.3% and 27.6% during the two periods, respectively). Interestingly enough, the number of publication by China rose from 5.8% in 2007-2011 to 9.5% in 2012-2016 period becoming the second country after the United States in publication in that field, overtaking the United Kingdom that had the second rank in 2007-2011 period. 

National Institute of Health (NIH) ranked first in funding the movement disorders research (6.6% to 5.3% during the two periods, respectively). Funding by the National Natural Science Foundation of China, the second funding agency, increased dramatically from 1.0% to 3.2% between the two periods.

Top five affiliated university in both time periods were University College London (UCL), Harvard University, University of Toronto, Mayo clinic, and Colombia University.

Regarding citation classic publications, between 2012 and 2016, 87 papers were cited more than 100 times and only one cited more than 400 times. The Nature (n = 17), Movement Disorders Journal (n = 7), Lancet (n = 6), Annals of Neurology (n = 6), and Neurons (n = 5) led in publishing these papers. 

The most frequently cited paper in movement disorders area entitled “Pathological Alpha-Synuclein Transmission Initiates Parkinson-Like Neurodegeneration in Nontransgenic Mice” by Luk, Kelvin C et al. (7 paper), was published in Science in 2012 and cited 439 times.

## Discussion

The findings show that during the past decade, the total number of publications in movement disorders field is increasing. The clinical aspect of neurology remains the most interested-in in movement disorders.

Journals such as the Movement Disorder Journal that tend to have a specific niche and specialize in sub-fields, publish most of the papers; but it seems that the trend in publishing on the open source journals, like PLOS one, is robust, and publication in such arenas has increased significantly in the past decade.

Although the United States is leading in publication in movement disorder, China is catching up allocating more funds to research in this field, and its scientific production is on the rise. The NIH and Chinese government are emerging as the major funding sources in this competition that in essence proves to be a welcoming collaboration of scientists beyond borders. 

Finally, it should be emphasized that the research landscape that bibliometric analysis shows is a very dynamic process and subject to change over time.

## Conclusion

To conclude, our study shows that the movement disorder is a dynamic and rapidly growing filed in neurology, and bibliographic analysis could be helpful in opening future research landscapes.

## Notes:

**How to cite this article:** Shafiei K, Khazaneha M. A bibliometric study of the movement disorder field by analyzing classic citation data on publication. Iran J Neurol 2018; 17(1): 53-5.

## References

[1] Allen L, Jones C, Dolby K, Lynn D, Walport M (2009). Looking for landmarks: The role of expert review and bibliometric analysis in evaluating scientific publication outputs. PLoS One.

[2] Patterson MS (2009). Are higher quality papers cited more often?. Phys Med Biol.

[3] Furlan JC, Fehlings MG (2006). A Web-based systematic review on traumatic spinal cord injury comparing the "citation classics" with the consumers' perspectives. J Neurotrauma.

[4] Seglen PO (1989). Use of citation analysis and other bibliometric methods in evaluation of the quality of research. Tidsskr Nor Laegeforen.

[5] Taubes G (1993). Measure for measure in science. Science.

[6] van Eck NJ, Waltman L, van Raan AF, Klautz RJ, Peul WC (2013). Citation analysis may severely underestimate the impact of clinical research as compared to basic research. PLoS One.

[7] Benito-Leon J, Louis ED (2013). The top 100 cited articles in essential tremor. Tremor Other Hyperkinet Mov (N Y).

[8] Mula M (2016). New trends and hot topics in epileptology: An analysis of top articles published in Epilepsy & Behavior in 2015. Epilepsy Behav.

[9] Li T, Ho YS, Li CY (2008). Bibliometric analysis on global Parkinson's disease research trends during 1991-2006. Neurosci Lett.

[10] Mariam N, Cavanna AE (2012). The most cited works in Tourette syndrome. J Child Neurol.

